# Large conductance Ca^2+^‐activated K^+^ (BK) channels promote secretagogue‐induced transition from spiking to bursting in murine anterior pituitary corticotrophs

**DOI:** 10.1113/jphysiol.2015.284471

**Published:** 2015-01-23

**Authors:** Peter J. Duncan, Sevgi Şengül, Joël Tabak, Peter Ruth, Richard Bertram, Michael J. Shipston

**Affiliations:** ^1^ Centre for Integrative PhysiologyCollege of Medicine and Veterinary MedicineUniversity of EdinburghEdinburghEH8 9XDUK; ^2^ Department of MathematicsFlorida State UniversityTallahasseeFL 32306USA; ^3^ Division of PharmacologyToxicology and Clinical PharmacyInstitute for PharmacyUniversity of TübingenD‐72076TübingenGermany

## Abstract

**Key points:**

- Corticotroph cells of the anterior pituitary are electrically excitable and are an integral component of the hypothalamic‐pituitary‐adrenal axis which governs the neuroendocrine response to stress.
- Corticotrophs display predominantly single spike activity under basal conditions that transition to complex bursting behaviours upon stimulation by the hypothalamic secretagogues corticotrophin‐releasing hormone (CRH) and arginine vasopressin (AVP); however, the underlying mechanisms controlling bursting are unknown.
- In this study, we show that CRH and AVP induce different patterns of corticotroph electrical activity, and we use an electrophysiological approach combined with mathematical modelling to show the ionic mechanisms for these differential effects.
- The data reveal that secretagogue‐induced bursting is dependent on large conductance Ca^2+^‐activated K^+^ (BK) channels and is driven primarily by CRH whereas AVP promotes an increase in single‐spike frequency through BK‐independent pathways involving activation of non‐selective cation conductances.
- As corticotroph excitability is differentially regulated by CRH and AVP this may allow corticotrophs to respond appropriately to different stressors.

**Abstract:**

Anterior pituitary corticotroph cells are a central component of the hypothalamic‐pituitary‐adrenal (HPA) axis essential for the neuroendocrine response to stress. Corticotrophs are excitable cells that receive input from two hypothalamic secretagogues, corticotrophin‐releasing hormone (CRH) and arginine vasopressin (AVP) to control the release of adrenocorticotrophic hormone (ACTH). Although corticotrophs are spontaneously active and increase in excitability in response to CRH and AVP the patterns of electrical excitability and underlying ionic conductances are poorly understood. In this study, we have used electrophysiological, pharmacological and genetic approaches coupled with mathematical modelling to investigate whether CRH and AVP promote distinct patterns of electrical excitability and to interrogate the role of large conductance calcium‐ and voltage‐activated potassium (BK) channels in spontaneous and secretagogue‐induced activity. We reveal that BK channels do not play a significant role in the generation of spontaneous activity but are critical for the transition to bursting in response to CRH. In contrast, AVP promotes an increase in single spike frequency, a mechanism independent of BK channels but dependent on background non‐selective conductances. Co‐stimulation with CRH and AVP results in complex patterns of excitability including increases in both single spike frequency and bursting. The ability of corticotroph excitability to be differentially regulated by hypothalamic secretagogues provides a mechanism for differential control of corticotroph excitability in response to different stressors.

**Abbreviations**
ACTH: adrenocorticotrophic hormone
AVP: arginine vasopressin
BF: burstiness factor
BK channel: large conductance Ca^2+^‐ and voltage‐activated K^+^ channel
BK‐far: BK channels located distantly from voltage‐gated Ca^2+^ channels
BK‐near: BK channels in close proximity to voltage‐activated Ca^2+^ channels
CRH: corticotrophin‐releasing hormone
GFP: green fluorescent protein
HPA: hypothalamic‐pituitary‐adrenal
IK channel: intermediate conductance Ca^2+^‐activated K^+^ channel
IP_3_: inositol trisphosphate
NMDG: *N*‐methyl‐d‐glucamine
PKA: protein kinase A
PKC: protein kinase C
POMC: proopiomelanocortin
STREX: stress regulated exon
ZERO: BK channels lacking STREX insert

## Introduction

Excitable cells, such as neurons and endocrine cells, exhibit diverse patterns of spontaneous electrical activity that can be modified in response to neuropeptide stimulation. These diverse responses are coordinated by an eclectic array of ion channels and establishing the role of any particular ion channel remains a significant challenge.

Endocrine cells of the anterior pituitary typically display spontaneous activity (Stojilkovic *et al*. 2010) that is characterised by either single action potentials or bursts of electrical activity, termed ‘pseudo‐plateau bursting’, that results in small oscillations of the membrane potential during the active phase of the burst, rather than full spikes (Tsaneva‐Atanasova *et al*. 2007; Stern *et al*. 2008; Vo *et al*. 2014). Pseudo‐plateau bursting has been proposed to increase intracellular Ca^2+^ to a greater extent than spiking alone which is thought to be important in driving hormone secretion in endocrine cells (Van Goor *et al*. 2001 *b*; Stojilkovic *et al*. 2005). Previous studies have identified a role for large conductance Ca^2+^‐ and voltage‐activated potassium (BK) channels in the generation of *spontaneous* pseudo‐plateau bursting in anterior pituitary somatotrophs and lactotrophs (Van Goor *et al*. 2001 *a*; Tsaneva‐Atanasova *et al*. 2007; Tabak *et al*. 2011) whereas gonadotrophs, that express little BK current, show spontaneous single spiking (Van Goor *et al*. 2001 *b*,*c*; Stojilkovic *et al*. 2010).

However, anterior pituitary corticotrophs, the central hub of the hypothalamic‐pituitary‐adrenal (HPA) axis which governs the homeostatic response to stress, express BK channels (Shipston & Armstrong, 1996; Shipston *et al*. 1999; Tsaneva‐Atanasova *et al*. 2007; Brunton *et al*. 2007; Stern *et al*. 2008; Liang *et al*. 2011; Vo *et al*. 2014) yet display predominantly spontaneous single spike activity but can *transition* to pseudo‐plateau bursting when stimulated (Kuryshev *et al*. 1997; Van Goor *et al*. 2001 *b*; Stojilkovic *et al*. 2005; Liang *et al*. 2011). This suggests that under unstimulated conditions BK channels in corticotrophs do not contribute to spontaneous electrical activity but may promote pseudo‐plateau bursting in response to the hypothalamic secretagogues corticotrophin‐releasing hormone (CRH) and arginine vasopressin (AVP). CRH and AVP activate distinct G‐protein receptor signalling cascades: the cAMP/protein kinase A (PKA) and inositol trisphosphate (IP_3_)/protein kinase C (PKC) pathways, respectively (Antoni, 1986; King & Baertschi, 1990; Van Goor *et al*. 2001 *a*; Tsaneva‐Atanasova *et al*. 2007; Tabak *et al*. 2011), both of which have been reported to control BK channel activity and properties in pituitary cells, including corticotrophs (Shipston *et al*. 1996; Tian *et al*. 2008; Zhou *et al*. 2012 *b*).

Fast activating BK channels (BK‐near), in close proximity to voltage‐activated calcium channels, have been demonstrated to facilitate pseudo‐plateau bursting (Tabak *et al*. 2011). The rapid activation of the channels that occurs during the upstroke of an action potential limits the spike amplitude and activation of delayed rectifier K^+^ channels and thus allows the membrane potential to oscillate, resulting in a burst (Tabak *et al*. 2011; Vo *et al*. 2014). BK channels that are located distantly from voltage‐gated Ca^2+^ channels (BK‐far) are responsible for the termination of a burst (Tsaneva‐Atanasova *et al*. 2007). While the molecular basis for these different populations is not established, importantly functional diversity of BK channel properties in anterior pituitary cells can be conferred by multiple mechanisms including alternative pre‐mRNA splicing and post‐translational modification of the pore‐forming subunit encoded by a single gene, *KCNMA1* (for example see Tian *et al*. 2004, 2008; Chen *et al*. 2005; Stojilkovic *et al*. 2010).

In this study, we have developed a mathematical model of corticotrophs and used this in conjunction with pharmacological and genetic approaches to interrogate the role of BK channels in spontaneous and CRH/AVP‐evoked electrical activity in native male mouse corticotrophs. We reveal that corticotroph BK channels do not play a significant role in spontaneous electrical activity but are essential for the transition to bursting upon CRH stimulation. Furthermore, we reveal that AVP, in contrast to CRH, does not promote bursting, but rather increases the frequency of single spikes and is independent of functional BK channels. Thus CRH and AVP engage BK channel‐dependent and ‐independent pathways, respectively, and BK channels are essential for the CRH‐induced transition to pseudo‐plateau bursting in murine corticotrophs.

## Methods

### Animals

Mice lacking the pore‐forming exon of the BK channel α‐subunit (BK^−/−^ mice; Sausbier *et al*. 2004) were backcrossed for at least 10 generations with mice expressing green fluorescent protein (GFP) under the proopiomelanocortin (POMC) promoter (Pinto *et al*. 2004) to generate BK‐POMC‐GFP mice with a SV129/C57BL6 mixed background. More than 99% of GFP‐positive cells also stain for ACTH in our assays and thus all GFP‐positive cells of the anterior pituitary are corticotrophs and lack any functional BK channels in the BK^−/−^ background. The genotype of all animals used was verified for each pituitary isolation and culture generated. The mice show normal gross pituitary morphology and the same number of corticotrophs although pituitary ACTH content, but not POMC mRNA, is suppressed as reported previously for the BK^−/−^ mice (Brunton *et al*. 2007) compared to their littermate (wild‐type, WT) controls. Mice were caged in groups of two to four under standard laboratory conditions (lights on at 07.00 h, lights off at 19.00 h, 21ºC, with tap water and chow available *ad libitum*). WT mice or mice deficient for the BK channel (BK^−/−^) were used from the same litters generated by a cross of mice heterozygous for the BK allele. Male mice, aged 2–5 months, were used for pituitary cell culture with tissue collection performed between 08.30 h and 10.00 h in accordance with UK Home Office requirements (PPL 60/4349) and University of Edinburgh ethical review committee approval.

### Pituitary cell culture

Three to four mice were killed by cervical dislocation and the pituitaries removed and cut to remove the intermediate (which contain POMC‐expressing melanotrophs) and posterior lobes. The remaining anterior lobe was chopped by hand with a single‐edged razor blade in two directions (pituitary rotated by 90 deg). The tissue was digested in a solution of Dulbecco's modified Eagle's medium (DMEM; Invitrogen Ltd., Paisley, UK) containing 25 mm Hepes, 0.25% trypsin (Worthington Biochemical Corp., Lakewood, NJ, USA) and 10 μg ml^−1^ DNase I (Sigma‐Aldrich Company Ltd., Gillingham, UK) for 20 min in a 37ºC water bath. The tube was shaken every 5 min to ensure a complete and even digestion. Following digestion, the tissue was allowed to settle to the bottom and the supernatant aspirated. One millilitre of inhibition solution (DMEM containing 0.5 mg ml^−1^ soybean trypsin inhibitor, 100 kallikrein units aprotinin (200× dilution of Sigma stock), 10 μg ml^−1^ DNase I) was added and triturated using a Gilson P1000 Pipetman (Gilson U.K., Luton, Bedfordshire, UK) set to 1 ml (∼40 times). A further 4 ml of inhibition solution was added and the resulting cell suspension was filtered through a pre‐wetted 70 μm nylon mesh (BD Bioscience, Oxford, UK) and diluted with an equal volume of culture media (DMEM containing 25 mm Hepes, 5 μg ml^−1^ insulin, 50 μg ml^−1^ transferrin, 30 nm sodium selenite, 0.3% bovine serum albumin (BSA; w/v), 4.2 μg ml^−1^ fibronectin and antibiotic/antimycotic (100× dilution of Sigma stock) and spun in a centrifuge at 100 *g* for 10 min. The supernatant was carefully removed and the cells were gently triturated with 1 ml of culture medium (∼40 times). The cell suspension was diluted appropriately with culture medium and plated on 12 mm coverslips in a six‐well plate (four coverslips per well) and incubated at 37ºC in 5% CO_2_. The medium was replaced every 2 days with an antibiotic/antimycotic‐free medium (DMEM containing 25 mm Hepes, 5 μg ml^−1^ insulin, 50 μg ml^−1^ transferrin, 30 nm sodium selenite, 0.3% BSA (w/v) and 4.2 μg ml^−1^ fibronectin) and electrophysiological recordings were obtained from cells 24–96 h post‐isolation. Over this culture period, cells displayed a typically simple stellate or ovoid morphology with no significant difference in behaviour or response to CRH or AVP (Bachem AG, Bubendorf, Switzerland) between days in culture.

### Electrophysiology

Current clamp electrophysiological recordings were obtained from corticotroph cells using the perforated patch mode of the whole‐cell patch clamp technique. The pipette solution contained amphotericin B at a concentration of 150 μg ml^−1^ and resulted in access resistances typically <40 MΩ within 10–20 min, which allowed stable recordings in excess of 20 min. The standard bath (extracellular) solution contained (in mm): 140 NaCl, 5 KCl, 2 CaCl_2_, 0.1 MgCl_2_, 10 Hepes and 10 glucose. The pH was adjusted to 7.4 with NaOH, osmolarity 300 mosmol l^−1^. The standard pipette (intracellular) solution contained (in mm): 10 NaCl, 30 KCl, 60 K_2_SO_4_, 1 MgCl_2_, 10 Hepes, 10 glucose and 50 sucrose. The pH was adjusted to 7.2 with KOH, 290 mosmol l^−1^.

Electrophysiological recordings were performed at room temperature (18–22°C) using Clampex 10.1 (Molecular Devices, Sunnyvale, CA, USA) with a sampling rate of 10 kHz and filtered at 2 kHz. Patch pipettes were fabricated from borosilicate glass (King Precision Glass, Inc., Claremont, CA, USA) using a Model P‐97 micropipette puller (Sutter Instrument Company, Novato, CA, USA). Pipette tips were heat polished and had resistances typically between 2 and 3 MΩ. Cell capacitance of corticotroph cells ranged from 2 to 10 pF and compensated series resistance was typically <20 MΩ. Drugs were applied to cells using a gravity perfusion system with a flow rate of 1–2 ml min^−1^ to minimise flow‐induced artefacts.

### Electrophysiology analysis

Current‐clamp recordings were performed using a standard 20 min protocol unless stated otherwise with analysis performed using Clampfit v.10.1 (Molecular Devices). Basal activity was recorded for 7 min before exposing cells to CRH and/or AVP for 3 min, concluding with a 10 min washout period. Activity of corticotroph cells was measured in 3 min blocks corresponding to basal activity (4–7 min), CRH/AVP‐evoked activity (10–13 min) and washout period (17–20 min). Consistent with previous studies, stimulation of corticotroph cells with CRH and AVP (0.2 nm and 2 nm, respectively) resulted in a robust membrane depolarisation (Liang *et al*. 2011). To account for this, during event analysis the baseline was adjusted relative to the depolarisation so that all events were measured relative to current membrane potential. Measurements were made of membrane potential, event frequency, and mean event duration. An event was defined from the point it reached threshold (Δ25 mV from baseline) until it fell below a re‐arm level (Δ5 mV). In addition to mean event duration, bursting behaviour was quantified through the calculation of a ‘burstiness factor’ (BF). This method classifies any event <100 ms as a spike and events >100 ms as a burst; a burstiness factor is calculated as the fraction of events that are bursts (Van Goor *et al*. 2001 *b*,*c*; Tabak *et al*. 2011).

### Mathematical model

The Hodgkin–Huxley formalism is used (Hodgkin & Huxley, 1952) with currents that are present in pituitary corticotrophs. In our model, the potential difference across the plasma membrane varies according to:

(1) CmdVdt=−(I Ca +IK‐ dr +I BK ‐ near +I BK ‐ far +IK‐ ir +I NS +I noise )

where *C*_m_ is the membrane capacitance. There are six ionic currents in the model as shown in Fig. 1 *A*. *I*_Ca_ is the high voltage‐activated dihydropyridine‐sensitive L‐type Ca^2+^ current that is responsible for most Ca^2+^ entry during an action potential. *I*_K‐dr_ is the rapidly activated delayed rectifier K^+^ current that is largely responsible for the downstroke of an action potential. The model also contains large‐conductance, voltage‐ and Ca^2+^‐activated K^+^ channels (BK channels). Some are located near Ca^2+^ channels and respond to Ca^2+^ in microdomains at open Ca^2+^ channels, producing the current *I*_BK‐near_. Others are situated away from Ca^2+^ channels and respond to the mean cytosolic Ca^2+^ concentration, producing the current *I*_BK‐far_. BK‐near channels represent stress‐regulated exon (STREX)‐type channels, while BK‐far channels represent BK channels lacking STREX insert (ZERO)‐type channels (Shipston *et al*. 1999; Chen *et al*. 2005; Zhou *et al*. 2012) although the spatial distribution of these channel variants, or any of the channels involved in excitability in corticotrophs, is not known. *I*_K‐ir_ is the barium‐insensitive inward rectifier K^+^ current that activates under hyperpolarisation. Also, the model has a current produced by non‐selective‐cation channels, *I*_NS_. The effect of system noise is included in the model through the current *I*_noise_.

**Figure 1:**
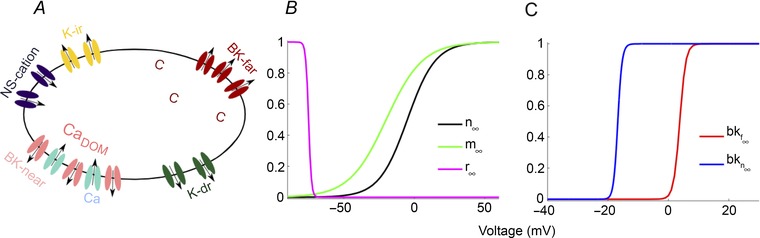
**Mathematical model of corticotroph cells** *A*, schematic diagram of the ionic currents in the pituitary corticotroph model. Ca_DOM_ is the free Ca^2+^ concentration in a microdomain and c is the mean free cytosolic Ca^2+^ concentration. Steady‐state activation functions for the K^+^ (black), L‐type Ca^2+^ (green) and K‐ir (magenta) channels (*B*) and for the BK‐far (red) and BK‐near (blue) channels (*C*).

The non‐selective‐cation current has constant conductance in our model I NS V=g NS V−V NS . The voltage‐dependent currents with dynamic conductances (*g*) are as follows:

(2) I Ca V=g Ca m∞VV−V Ca

(3) IK‐ dr V=gKnV−VK

(4) IK‐ ir V=gK‐ ir r∞VV−VK

(5) I BK ‐ far V,c=g BK ‐ far bkfV−VK

(6) I BK ‐ near V,c dom =g BK ‐ near bknV−VK

We assume that the Ca^2+^ channels and inward rectifying K^+^ channels activate instantaneously. The gating variable for the activation of delayed rectifier K^+^ current (*n*) has first order kinetics and changes with time according to

(7) $$\frac{dn}{dt}=\frac{n_{\infty }\left(V\right)-n}{\tau_{n}}.$$

The steady‐state functions are

(8) $$x_{\infty }\left(V\right)=\frac{1}{1+e^{\left(\frac{v_{x}-V}{s_{x}}\right)}},\;x=n,m,r.$$

For the near and far populations of BK channels we use the model of (Tsaneva‐Atanasova *et al*. 2007) for pituitary somatotrophs. The gating variables are

(9) db kndt=bkn∞V,c dom −bknτbkn

(10) db kfdt=bkf∞V,c−bkfτbkf

where *c*_dom_ is the free Ca^2+^ concentration in a microdomain and *c* is the mean free cytosolic Ca^2+^ concentration. The equilibrium functions are:

(11) bkn∞V,c dom =11+ exp −V−V bk ‐ near c dom k bk ,

(12) bkf∞V,c=11+ exp −V−V bk ‐ far ck bk .

where

(13) V BK ‐ near c dom =VBK0−k shift  ln c dom kCa BK ‐ near ,

(14) V BK ‐ far c=VBK0−k shift  ln ckCa BK ‐ far

and domain Ca^2+^ is modelled as proportional to the Ca^2+^ current:

c dom =−AI Ca V.

The equation for the mean intracellular Ca^2+^ concentration is

dcdt=−fαI Ca +kcc

where *f*is the fraction of free Ca^2+^ in the cytosol, α converts current to concentration and *k*_c_ is the Ca^2+^ pump rate.

The current due to channel noise is I noise =σNω where σ_N_is noise amplitude and ω is a Wiener variable. The model is implemented in the XPPAUT software program (Ermentrout, 2002) using the Euler method (d*t* = 0.1 ms), and the computer code is available for free download from http://www.math.fsu.edu/∼bertram/software/pituitary.

Parameters of the model are listed in Table 1 and the steady‐state activation functions for the currents are shown in Fig. 1 *B* and *C*.

**Table 1:** **Parameter values for mathematical model of corticotroph excitability**

Parameter	Value	Parameter	Value
*g*_Ca_	1.8 nS (basal), 2.2 nS (CRH)	kCa BK ‐ near	18 μm (basal), 6 μm (CRH)
*g*_NS_	0.1 nS (basal), 0.2 nS (AVP)	kCa BK ‐ far	6 μm
*g*_K_	8.2 nS	*k*_bk_	3 mV
$g_{K\text{‐}\mathrm{ir}}$	1 nS	*s_m_*	10
g BK ‐ near	2 nS	*s_n_*	10
g BK ‐ far	1 nS	*s_r_*	−1
*V*_Ca_	60 mV	$V_{BK_{0}}$	0.1 mV
*V*_NS_	−10 mV	*k*_shift_	18
*V*_K_	−75 mV	*A*	0.15
$v_{r}$	−60 mV	$k_{c}$	0.12 μm
$v_{m}$	−20 mV	*f*	0.01
$v_{n}$	−5 mV	α	0.0015
$\tau_{bk_{n}}$	20 ms (basal), 4 ms (CRH)	σ_N_	5 pA
$\tau_{bk_{f}}$	4 ms	*C*_m_	6 pF
$\tau_{n}$	40 ms		

### Statistics

The data were expressed as mean ± SEM (standard error of the mean), *n* = number of independent experiments. Statistical analysis was performed as appropriate by Student's *t* test and ANOVA analysis with the Bonferroni–Holm *post hoc* test (Microsoft Excel). Significant differences between groups were defined at *P < 0.05, **P < 0.01 and ***P < 0.001.

## Results

### CRH/AVP increases frequency and bursting in corticotroph cells

In corticotrophs isolated from POMC‐GFP male mice spontaneous activity was observed in more than 90% of cells recorded under current clamp using the perforated patch‐clamp approach, as previously observed in female corticotrophs (Liang *et al*. 2011) with predominantly large amplitude single‐spike action potentials (Fig. 2 *A* and *B*). Very occasional longer duration bursts of activity were observed, together with single spike action potentials, in less than 6% of all corticotrophs analysed under basal conditions. Corticotrophs had a resting membrane potential of −53.7 ± 1.5 mV, displayed spontaneous action potentials at a frequency of 0.34 ± 0.14 Hz and had a mean cell capacitance of 4.43 ± 0.94 pF (Fig. 2 *D* and *E*). The mean event duration of 69 ± 26 ms and a burstiness factor (BF) of 0.18 ± 0.10 are both consistent with predominantly single‐spike action potential behaviour (Fig. 2 *F* and *G*) in the unstimulated state. Replacement of external sodium ions with the large organic cation *N*‐methyl‐d‐glucamine (NMDG^+^) resulted in a significant (*P* = 0.00059) hyperpolarisation of 22.7 ± 2.8 mV (*n* = 5) within 1 min, accompanied by a cessation of spontaneous activity which was fully reversible following washout. These data are consistent with those previously observed in female mouse corticotrophs and that a background sodium conductance is important for setting the resting membrane potential of corticotrophs, as in other pituitary cells (Liang *et al*. 2011; Tomic *et al*. 2011).

**Figure 2:**
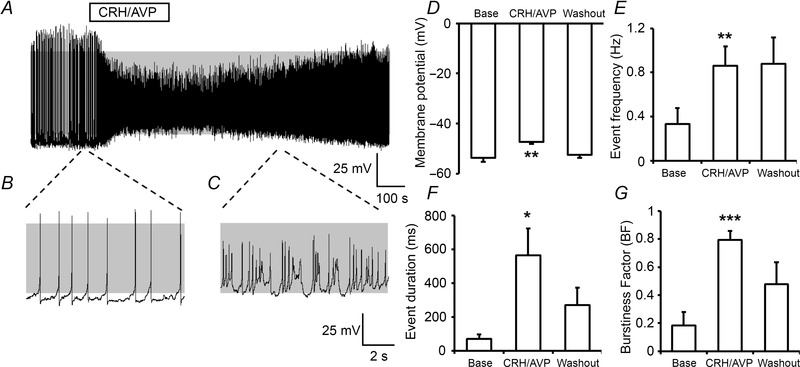
**Stimulation of corticotrophs with CRH/AVP** *A*, representative current‐clamp recording of a corticotroph cell exposed to 0.2 nm CRH and 2 nm AVP for 3 min. Under basal conditions corticotroph cells display predominantly single‐spike action potentials (*B*) which transition to complex bursting patterns following CRH/AVP exposure (*C*). Grey shading indicates membrane potential between −50 mV and +10 mV. Summary bar graphs illustrating that stimulation with CRH/AVP results in a membrane depolarisation (*D*) coupled with an increase in event frequency (*E*), event duration (*F*) and burstiness factor (BF; *G*). Data are means ± SEM, (*n* = 7 per group). *P < 0.05, **P < 0.01, ***P < 0.001, Student's *t* test compared to base values.

*In vivo*, corticotrophs are exposed to pulses of both CRH and AVP released from the hypothalamus in response to stress, resulting in the release of ACTH. Thus corticotrophs were stimulated with CRH and AVP (0.2 and 2 nm, respectively) at concentrations chosen to be physiologically relevant and within peak concentrations in portal circulation in response to stress (Gibbs & Vale, 1982; Sheward & Fink, 1991). A 3 min exposure to CRH/AVP resulted in a robust depolarisation and an increase in firing frequency (Fig. 2 *A*) with a transition from predominantly single‐spike action potentials (Fig. 2 *B*) to complex bursting patterns (Fig. 2 *C*) that included both single spike and ‘pseudo‐plateau burst’ behaviours. Following CRH/AVP exposure, there was a significant (*P* = 0.0031) membrane depolarisation to −47.4 ± 0.8 mV (Fig. 2 *D*) and a significant (*P* = 0.0080) increase in event frequency to 0.86 ± 0.18 Hz (Fig. 2 *E*). This was accompanied by a significant (*P* = 0.016) increase in mean event duration (564 ± 160 ms) following CRH/AVP stimulation (Fig. 2 *F*) as well as a significant (*P* = 0.00014) increase in the burstiness factor to 0.79 ± 0.06 (Fig. 2 *G*), indicating a switch to predominantly pseudo‐plateau bursting following CRH/AVP exposure. After 10 min washout of CRH/AVP, membrane potential returned to −52.5 ± 1.3 mV which was not significantly different to baseline (Fig. 2 *D*). However, corticotroph activity remained elevated compared to pre‐stimulation with event frequency elevated at 0.88 ± 0.2 Hz (Fig. 2 *E*). Although event duration and the burstiness factor were elevated compared to pre‐stimulation levels, they were suppressed compared to levels 3 min following CRH/AVP application (Fig. 2 *F* and *G*). These data reveal that exposure of corticotrophs to physiological levels of the hypothalamic secretagogues CRH and AVP result in complex patterns of excitability that include increases in event frequency and a transition to pseudo‐plateau bursting.

### Modelling of the CRH/AVP effect in pituitary corticotrophs

To examine whether our mathematical model of the corticotroph can recapitulate the key features of both spontaneous (basal) activity and the subsequent depolarisation and transition to bursting following CRH and AVP stimulation we changed ionic current parameters in the model that correspond to currents implicated in the control of corticotroph excitability. The model consists of six ionic currents (Fig. 1 *A* and *B*): L‐type Ca^2+^ current (*I*_Ca_), delayed rectifier K^+^ current (*I*_K‐dr_), inward rectifier K^+^ current (*I*_K‐ir_), BK‐near (*I*_BK‐near_) and BK‐far (*I*_BK‐far_) currents and non‐selective current (*I*_NS_) with a simple geometry as the spatial organisation of ion channels and Ca^2+^ pools in corticotrophs is very poorly characterised. See Methods for full description.

L‐type Ca^2+^ channels are critical for CRH/AVP‐stimulated ACTH secretion and CRH has been reported to increase L‐type Ca^2+^ current in a variety of corticotroph models (Mollard *et al*. 1987; Guérineau *et al*. 1991; Kuryshev *et al*. 1995). CRH and AVP have been reported to activate non‐selective cation channels (Takano *et al*. 1996; Mani *et al*. 2009) in corticotrophs or other cells and a non‐selective Na^+^‐dependent conductance is critical for determining the resting membrane potential (Liang *et al*. 2011). Although CRH has been reported to regulate inward rectifier K^+^ current *I*_K‐ir_ in rat corticotrophs (Kuryshev *et al*. 1997) the barium‐sensitive *I*_K‐ir_ has no functional effect in mouse corticotrophs (Liang *et al*. 2011). Distinct BK channel splice variants, including the stress regulated exon (STREX) and ZERO (lacking STREX insert) splice variants of the channel are expressed in the anterior pituitary and murine corticotrophs (Shipston & Armstrong, 1996; Shipston *et al*. 1999; Brunton *et al*. 2007; Liang *et al*. 2011). The model incorporates two distinct BK channel populations: BK‐near and BK‐far. While the molecular identity of these populations is not prescribed, the characteristics of these populations are similar to those of the STREX and ZERO variants, respectively. For example, BK‐near (STREX) have a significantly left‐shifted apparent voltage for half‐maximal activation compared to BK‐far (ZERO) channels (Xie & McCobb, 1998; Chen *et al*. 2005) (Fig. 1 *C*). Moreover, activation of the cAMP/PKA pathway shifts the properties of STREX channels to channels with ZERO‐like properties including a significant right shift in the half‐maximal voltage of activation and faster activation kinetics (Tian *et al*. 2001 *a*, 2004; Chen *et al*. 2005; Zhou *et al*. 2012 *a*).

Thus in our model we first simulated the application of CRH/AVP through changes to three ionic currents: the L‐type Ca^2+^ current (*I*_Ca_), the non‐selective cation current (*I*_NS_), and the BK‐near current (*I*_BK‐near_). Figure 3 *A* and *B* shows the model corticotroph cell exhibiting spontaneous spiking over the first 100 s as seen experimentally. The slow spiking is due to the noise that pushes the voltage randomly above spike threshold with the resting membrane potential set close to threshold by *g*_NS_. The application of CRH/AVP is simulated by increasing the conductances *g*_NS_ and *g*_Ca_ (Table 1). Also, the BK‐near channel time constant $\tau_{bk_{n}}$ is decreased along with kCa BK − near  (Table 1), producing a faster activation of this current and shifting its activation curve rightward to overlap that of the BK‐far activation curve (Fig. 1 *C*, blue curve becomes like red curve). After these changes to the model parameters, the low frequency spiking changes to high‐frequency bursting (Fig. 3 *A* and *C*). Moreover, the spike amplitude decreases substantially (by ∼20 mV) and the base membrane potential increases (by ∼20 mV), as seen experimentally. The effects of drug washout is simulated by slowly changing parameters back to their original values (Fig. 3 *A*).

**Figure 3:**
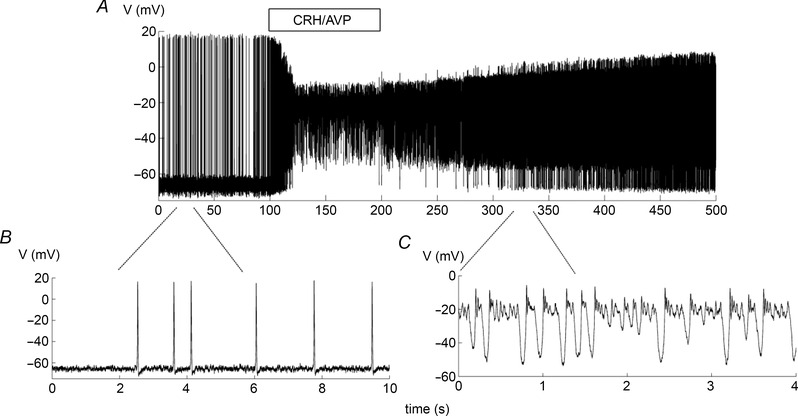
**Modelling the effects of CRH/AVP stimulation** Adding CRH/AVP transforms spontaneous spiking to bursting in the corticotroph model. The CRH/AVP effect is modelled as an increase in g_Ca_ from 1.8 nS to 2.2 nS, an increase in g_NS_ from 0.1 nS to 0.2 nS, a decrease in $\tau_{bk_{n}}$ from 20 ms to 4 ms and a decrease in kCa BK − near  from 18 μm to 6 μm.

### CRH and AVP differentially regulate corticotroph excitability *in vitro*

CRH and AVP act synergistically to increase ACTH secretion from corticotroph cells and activate distinct G‐protein‐coupled receptor‐activated intracellular signalling cascades: the cAMP/PKA and IP_3_/PKC pathways, respectively. Stimulation of corticotrophs with combined secretagogues CRH/AVP increased both firing frequency and bursting behaviour. Previous studies have reported that CRH and AVP are individually able to regulate electrical excitability in corticotrophs (e.g. see Mollard *et al*. 1987; Guérineau *et al*. 1991; Lee & Tse, 1997; Tse & Lee, 1998). We thus asked whether CRH and AVP, at physiologically relevant concentrations reported in the portal circulation (Gibbs & Vale, 1982; Sheward & Fink, 1991), promote distinct, or similar, patterns of electrical activity when applied alone in an attempt to dissect key conductances that may be the targets of these neuropeptides.

Individually, both CRH (Fig. 4 *A* and *B*) and AVP (Fig. 4 *C* and *D*) were still able to produce an increase in corticotroph excitability. However, the patterns of stimulated excitability were distinct. Treatment of corticotrophs with 0.2 nm CRH resulted in a significant (*P* = 0.0057) depolarisation from –53.0 ± 2.4 mV to –43.0 ± 3.8 mV. Firing frequency increased significantly (*P* = 0.043) from 0.13 ± 0.07 Hz to 1.45 ± 0.68 Hz (Fig. 4 *E*). Following 10 min washout, event frequency had significantly declined to 0.40 ± 0.22 Hz in contrast to the sustained elevation of excitability observed in cells treated with combined CRH/AVP (0.88 ± 0.24 Hz). A transition from spiking to bursting was observed in all tested cells and mean event duration increased significantly (*P* = 0.021) from 63 ± 49 ms to 378 ± 267 ms following CRH stimulation (Fig. 4 *F*). In contrast, cells treated with 2 nm AVP alone failed to produce a significant depolarisation, but there was a significant (*P* = 0.033) increase in firing frequency from 0.32 ± 0.17 Hz to 1.77 ± 0.42 Hz (Fig. 4 *E*). Firing frequency remained elevated (1.33 ± 0.60 Hz) following 10 min washout suggesting that AVP promotes a sustained increase in activity. Interestingly, AVP alone failed to induce a transition from spiking to bursting in any cell examined. Mean event duration was 55 ± 46 ms under basal conditions and did not significantly increase (105 ± 78 ms) following AVP exposure (Fig. 4 *F*). AVP has been reported to stimulate intracellular Ca^2+^ release, although most reports utilise supraphysiological AVP levels an order of magnitude greater than used here, which are reported to activate Ca^2+^‐activated potassium currents that produce brief hyperpolarisation in rat corticotrophs (Corcuff *et al*. 1993). We never see an AVP‐induced hyperpolarisation under our recording conditions suggesting that either AVP (2 nm) does not significantly promote intracellular Ca^2+^ release in our system or this calcium elevation is not efficiently coupled to activation of Ca^2+^‐activated potassium channels.

**Figure 4:**
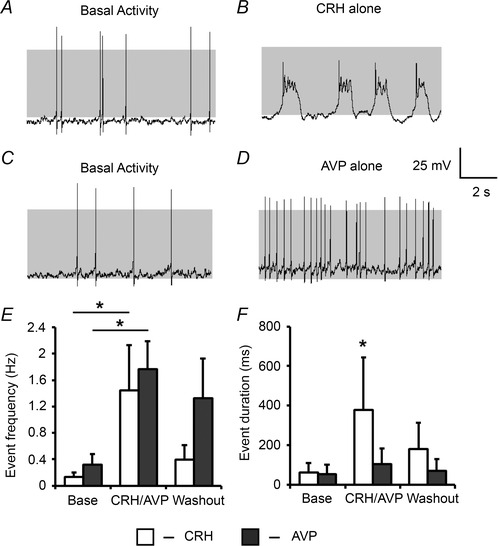
**CRH and AVP differentially regulate corticotroph excitability** Representative traces of corticotroph cells before (*A*) and following (*B*) 0.2 nm CRH alone or before (*C*) and after (*D*) exposure to 2 nm AVP for 3 min. Summary bar graphs reveal that individually CRH and AVP can both induce an increase in event frequency (*E*) but only CRH is able to produce an increase in event duration which corresponds to a transition to bursting behaviour (*F*). Data are means ± SEM (*n* > 3 per group). *P < 0.05 with ANOVA in *E*, and Mann–Whitney *U* test in *F*, compared to base values.

These data suggest that CRH can drive a transition to pseudo‐plateau bursting while AVP promotes a sustained increase in single‐spike frequency but does not support a transition to bursting. This predicts that CRH and AVP differentially control conductances important for increased electrical excitability in corticotrophs and that conductances regulated by the cAMP/PKA pathway are probably responsible for the transition to bursting.

### Distinct conductances differentially regulate spike frequency and bursting in the corticotroph model

The experimental findings we observed after the addition of CRH only or AVP only were further investigated in our model. In the experiments, adding only CRH (without AVP) changes the basal single‐spike activity to bursting (Fig. 4 *A* and *B*). Since CRH may act on several conductances, we asked which conductances were necessary for conversion to bursting. We found that increasing either the non‐selective‐cation current conductance *g*_NS_ or Ca^2+^ current conductance *g*_Ca_ increases spike frequency but does not produce bursting. However, making the BK‐near channels similar to BK‐far by reducing $\tau_{bk_{n}}$ of the BK‐near channel from 20 ms to 4 ms and right‐shifting its activation curve (kCa BK ‐ near  from 18 to 6 μm) is sufficient to convert spiking to bursting without the need to make any other changes (Fig. 5 *A* and *B*). A small additional increase of the L‐type conductance did not prevent that transition to bursting, but increased burst frequency and amplitude.

**Figure 5:**
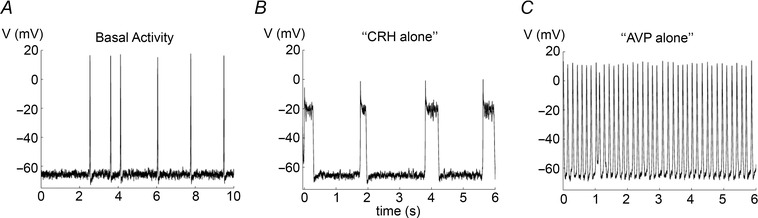
**Simulations of CRH and AVP alone** *A*, basal spiking activity. *B*, CRH alone transforms spiking to high frequency bursting due to changes in the currents *I*_Ca_ (*g*_Ca_ is increased from 1.8 nS to 2.2 nS) and *I*_BK‐near_ ($\tau_{bk_{n}}$ is decreased from 20 ms to 4 ms and kCa BK − near  is decreased from 18 μm to 6 μm)_._ *C*, AVP alone modulates *I*_NS_ (*g*_NS_ is increased from 0.1 nS to 0.2 nS) which leads to fast spiking.

The data in Fig. 4 *C* and *D* show that application of AVP alone greatly increases the spike frequency but does not convert spiking to bursting. We found that increasing *g*_NS_ alone (from 0.1 to 0.2 nS) is sufficient to generate the effect seen in experiments. That is, an increase in firing frequency and a slight depolarisation of the membrane potential without a transition to bursting (Fig. 5 *C*). In accordance with a role for *g*_NS_ in AVP action, step current injection alone only stimulates an increase in firing frequency, not a transition to bursting (for example, a 1 pA current injection results in a (2.41 ± 0.31)‐fold (*n* = 4) increase in action potential frequency). Moreover, inhibition of the non‐selective sodium conductance results in membrane hyperpolarisation and prevents CRH/AVP‐induced depolarisation and increase in excitability (Liang *et al*. 2011). Changes in *g*_Ca_ alone increases spike frequency and amplitude, while changes in $\tau_{bk_{n}}$ and kCa BK ‐ near  convert spiking to bursting. Hence the model suggests that changes in BK‐near cause the CRH‐induced transition to bursting, and an increase in the non‐selective cation conductance causes the increased spike frequency induced by AVP.

In the model, BK channels have almost no effect on the spontaneous low‐frequency spiking activity. Figure 6 *A* shows spontaneous activity in the absence of BK conductance. When application of CRH and AVP together is simulated the spike frequency greatly increases and the model cell is depolarised, but there is no bursting (Fig. 6 *B*). However, when even a small fraction (15%) of BK conductance remains there is a mixture of bursting and fast spiking (Fig. 6 *C*).

**Figure 6:**
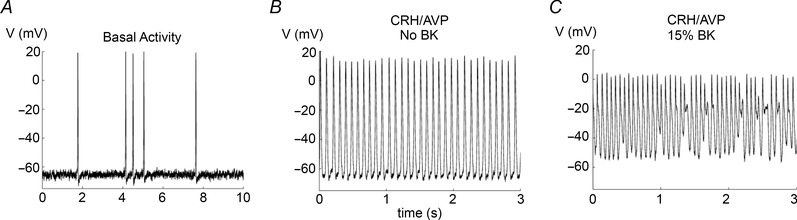
**Simulations with little or no BK conductance** *A*, basal spiking activity in the absence of BK conductance. *B*, CRH/AVP increases spike frequency without bursting when there is no BK conductance. *C*, when some BK conductance remains, CRH/AVP elicits some bursting mixed with fast spiking.

### CRH/AVP‐evoked bursting activity is suppressed by pharmacological inhibition of BK channels

The model predictions of Fig. 6 were tested in corticotrophs exposed to the selective BK channel inhibitor paxilline (1 μm). To investigate the role of BK channels in regulating basal activity, corticotrophs were exposed to paxilline for 3 min. In 3/3 cells tested, paxilline had no effects on spontaneous corticotroph activity for all parameters measured. These results are not surprising as the BK channel has been suggested to promote bursting behaviour which is uncommon under basal conditions.

To investigate the role of BK channels in the CRH/AVP response, corticotroph cells were treated with paxilline (1 μm) for 4 min prior to CRH/AVP exposure. Paxilline remained present throughout the remainder of the recording. In comparison to control cells, paxilline‐treated cells showed predominantly single‐spike action potentials under basal conditions (Fig. 7 *A*). In contrast, there was a significant reduction in CRH/AVP‐evoked bursting behaviour (Fig. 7 *B*). Under basal conditions, paxilline‐treated cells had a resting membrane potential of −54.2 ± 2.8 mV and a spontaneous event frequency of 0.27 ± 0.20 Hz (*n* = 7). Paxilline‐treated cells also had a mean event duration of 148 ± 39 ms and a burstiness factor of 0.23 ± 0.13. The basal properties of paxilline‐treated cells were not significantly different to untreated cells for all parameters measured.

**Figure 7:**
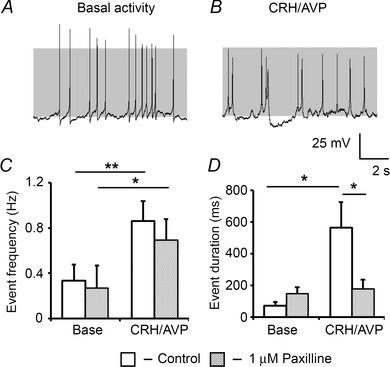
**Pharmacological blockade of BK channels reduces bursting activity** *A*, representative traces of corticotrophs pretreated with 1 μm paxilline, which reduces CRH/AVP‐evoked bursting behaviour (*B*). *C*, paxilline has no effect on the ability of CRH/AVP to increase event frequency but significantly reduces event duration (*D*). Data are means ± SEM (*n* = 7 per group). *P < 0.05, **P < 0.01, ANOVA compared to respective base values.

Following CRH/AVP exposure, there was a significant (*P* = 0.0068) depolarisation from −54.2 ± 2.8 mV to −46.3 ± 2.9 mV in paxilline‐treated cells which was no different from controls. Event frequency significantly (*P* = 0.044) increased to 0.69 ± 0.16 Hz (Fig. 7 *C*) which was not significantly different to untreated cells (0.86 ± 0.18 Hz). A transition to bursting was observed in only 4/8 cells after CRH/AVP stimulation, compared to 7/7 of control cells. CRH/AVP was unable to produce a significant increase in mean event duration (178 ± 58 ms) in paxilline‐treated cells (Fig. 7 *D*), which was significantly (*P* = 0.021) reduced compared to untreated cells (564 ± 160 ms). The burstiness factor following CRH/AVP stimulation was 0.43 ± 0.15 which was significantly (*P* = 0.018) reduced compared to untreated cells (0.79 ± 0.06).

### BK‐knockout mice show reduced bursting compared to wild‐types

Pharmacological blockade of BK channels resulted in a reduction in CRH/AVP‐evoked bursting activity, as predicted by the model. To further investigate the role of BK channels in promoting bursting, cells isolated from BK channel knockout mice (BK^−/−^) were exposed to CRH/AVP for 3 min following the same 20 min protocol. The mean cell capacitance of BK^−/−^ cells was 4.16 ± 0.40 pF (*n* = 6) which was not significantly different from wild‐type cells (4.89 ± 0.29 pF).

Current‐clamp recordings revealed 4/6 BK^−/−^ cells displayed spontaneous activity with 6/6 showing an increase in activity following CRH/AVP stimulation (Fig. 8 *A* and *B*). In comparison to paxilline‐treated cells, corticotrophs isolated from BK^−/−^ mice showed no difference in basal membrane potential (−53.7 ± 2.3 mV), frequency (0.40 ± 0.19 Hz), mean event duration (153 ± 106 ms) or burstiness factor (0.15 ± 0.11) compared to wild‐type cells (*n* = 6). CRH/AVP exposure resulted in a significant (*P* = 0.00074) depolarisation to −45.1 ± 2.4 mV which was not significantly different to wild‐type. CRH/AVP was able to induce a significant (*P* = 0.046) increase in firing frequency to 2.30 ± 0.79 Hz (Fig. 8 *C*). Interestingly, peak firing frequency was significantly (*P* = 0.040) elevated compared with controls (0.86 ± 0.18 Hz) representing a 2.7‐fold increase in CRH/AVP‐evoked activity. Following CRH/AVP stimulation, bursting activity was observed in 3/6 cells. Basal mean event duration was 153 ± 106 ms which did not significantly increase (305 ± 127 ms) following CRH/AVP (Fig. 8 *D*).

**Figure 8:**
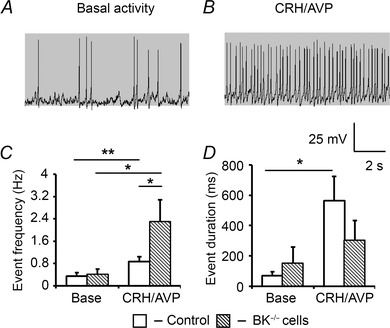
**Genetic deletion of BK channels reduces bursting activity** *A*, representative traces of corticotrophs isolated from BK^−/−^ mice which also show a reduction in bursting following CRH/AVP stimulation (*B*). BK^−/−^ cells show a higher event frequency following CRH/AVP compared to wild‐type cells (*C*) but show a decrease in event duration (*D*). Data are means ± SEM (*n* > 6 per group). *P  <0.05, **P  <0.01, ANOVA compared to respective base values.

These pharmacological and genetic data combined show that CRH/AVP is still able to produce a robust increase in electrical activity in BK^−/−^ cells. However, the increase in activity was largely associated with an increase in firing frequency rather than a transition to bursting supporting a key role for BK channels in the CRH/AVP‐induced transition to bursting.

## Discussion

In this paper we have exploited electrophysiological, pharmacological and genetic approaches in conjunction with mathematical modelling of anterior pituitary corticotroph cells to examine the mechanisms controlling secretagogue‐induced electrical excitability. Importantly, our data reveal that the hypothalamic neuropeptides CRH and AVP stimulate distinct patterns of electrical excitability. CRH promotes bursting behaviour that is dependent upon functional BK channels whereas AVP promotes an increase in firing frequency of single action potentials through BK‐independent pathways probably involving activation of non‐selective conductances. Thus, as distinct stressors can stimulate differential release of CRH and AVP and corticotroph excitability is differentially controlled by these two hypothalamic secretagogues, the pattern of corticotroph excitability may be distinct for different stressors.

In this study we examined corticotrophs from male mice expressing GFP under the control of the POMC promoter to aid visual identification. In agreement with previous studies in female mice (Liang *et al*. 2011) >90% of male corticotrophs displayed spontaneous activity with predominantly single action potentials that varied widely in frequency (0.01–1 Hz) in unstimulated conditions using the perforated patch‐clamp recording approach. In <6% of corticotrophs we observed very occasional longer bursts of activity alongside single spikes. Whether these infrequent longer events in the basal state reflect stochastic channel behaviour or reflect the activity of endogenous signalling pathways that control ion channel function, as has been reported for the cAMP‐signalling pathways in other pituitary cells (Kucka *et al*. 2013), remains to be determined. However, the predominant phenotype of unstimulated murine corticotrophs was single spike activity of variable frequency that did not differ over the 1–4 days in culture. A previous study in mouse corticotrophs reported that corticotrophs are not spontaneously active (Lee *et al*. 2011); however, this employed conventional whole‐cell recording that results in intracellular dialysis, a procedure that abolishes spontaneous activity in murine corticotrophs (Liang *et al*. 2011). The modelling, in conjunction with electrophysiological analysis, revealed that the resting membrane potential was largely determined by the non‐selective sodium conductance with single spike activity resulting from stochastic fluctuations that bring the voltage above spike threshold. In accordance with other anterior pituitary cell types that display single spike activity under basal conditions, spontaneous corticotroph activity was unaffected by pharmacological or genetic inhibition of the BK channel (Van Goor *et al*. 2001 *b*,*c*).

Stimulation of corticotrophs with physiological concentrations of CRH/AVP resulted in a robust depolarisation and increase in event frequency coupled with a transition from predominantly single‐spike action potentials to complex bursting patterns. The modelling analysis revealed that the transition to the complex bursting pattern following combined CRH/AVP could be explained primarily by changes in three conductances: the L‐type Ca^2+^ conductance, non‐selective cation conductance and the properties of the BK‐near conductance. Separately CRH and AVP are able to drive an increase in electrical activity of the corticotroph but differentially modulate the pattern of electrical activity. These distinct patterns result from the differential contribution of these three conductances in CRH and AVP‐stimulated excitability. Corticotrophs stimulated with CRH alone promoted a transition to bursting, an effect dependent upon changes that make the BK‐near channels like the BK‐far channels, i.e. a right‐shifted activation curve and faster activation. Thus regulation of BK‐near alone may control bursting, although regulation of *g*_Ca_ and *g*_NS_ are required for the significant depolarisation of resting membrane potential and increase in the frequency of spikes and bursts. Indeed, when BK channels are blocked pharmacologically, or deleted genetically, stimulation promotes an increase in single spike activity. In contrast, AVP stimulated an increase in single spike frequency without a transition to bursting, an effect dependent upon *g*_NS_ in the model. Intriguingly, AVP alone (or CRH/AVP) resulted in an increase in event frequency which remains elevated at the end of the washout period whereas with CRH alone activity declines during washout. This suggests that AVP prolongs the duration of the response to hypothalamic secretagogues and supports the hypothesis that CRH mediates a rapid increase in ACTH secretion whereas AVP acts to elicit a plateau phase (Lee & Tse, 1997; Tse & Lee, 1998). Clearly the ability of CRH and AVP to promote distinct patterns of electrical excitability, and consequently distinct patterns of Ca^2+^ dynamics in corticotrophs, is likely to be important for both short‐term control of ACTH secretion as well as longer‐term control of gene transcription and other Ca^2+^‐regulated mechanisms in corticotrophs. Important in this regard will be a greater understanding of the spatial distribution of the ion channels controlling excitability, intracellular Ca^2+^ stores and their relationship to secretory vesicle localisation and release. Cytological and ultrastructural analysis of corticotrophs *in situ* in rat has revealed heterogeneity in corticotroph morphologies including ‘stellate’ and ovoid cell types (e.g. see Yoshimura & Nogami, 1981; Childs, 1987). The proportion of these morphologically distinct corticotrophs differs between species (e.g. human corticotrophs tend to be ovoid or round) as well as during development and in response to environmental challenge. Based on electron microscopy analysis ACTH‐containing granules are typically localised toward the cell periphery and in a proportion of corticotrophs accumulate in extended processes. More recent analysis of the three‐dimensional architecture of murine corticotrophs from POMC‐GFP‐expressing mice also shown heterogeneity in corticotroph morphology as well as extensive anatomical networks *in situ* (Budry *et al*. 2011). Whether such morphologically distinct corticotrophs display distinct properties, regulation or function is not known. However, we saw no difference in behaviour or responses in the simple stellate and ovoid cells analysed in our short‐term cultures.

How may CRH and AVP control conductances required for stimulation of distinct patterns of electrical activity? CRH and AVP operate through two distinct intracellular signalling cascades activated via distinct G protein‐coupled receptors (Antoni, 1986; King & Baertschi, 1990; Stojilkovic *et al*. 2010). CRH exerts its actions exclusively through CRH receptor 1 in corticotroph cells resulting in an increase in cytosolic cAMP with subsequent activation of downstream effectors, predominantly PKA. In contrast, AVP acts through V1B receptors, and results in the cleavage of phosphatidylinositol 4,5‐bisphosphate (PIP_2_) to IP_3_ and diacyl glycerol, the latter leading to the activation of PKC. Voltage‐dependent Ca^2+^ influx via L‐type Ca^2+^ channels is essential for CRH‐ and AVP‐evoked ACTH secretion. CRH, via PKA, has been shown in a number of models to stimulate Ca^2+^ influx by activating L‐type Ca^2+^ channels, most likely via PKA phosphorylation of the channel (Kuryshev *et al*. 1996). Both CRH and AVP have been reported to activate non‐selective cation conductances (Takano *et al*. 1996; Mani *et al*. 2009), thus providing potential pathways for driving cellular depolarisation. Although the molecular identity of these non‐selective conductances has not been defined, work in other pituitary cell types suggest that it may, in part, be mediated via transient receptor potential‐like conductances that are also regulated by the cAMP/PKA pathway (Tomic *et al*. 2011).

Our data reveal that BK channels play an important role in CRH‐stimulated generation of bursting behaviour in corticotroph cells. Previous studies have identified a correlation between the level of BK expression in anterior pituitary cell types and the incidence of spontaneous bursting activity (2001Van Goor *et al*. 2001*a*,*c*). Furthermore, previous studies have suggested that there exist two populations of BK channels involved in the generation of bursting (Tsaneva‐Atanasova *et al*. 2007). BK channels located in close proximity to voltage‐gated Ca^2+^ channels (BK‐near) are important in the generation of a burst whereas BK channels located distantly from Ca^2+^ channels (BK‐far) are required for burst termination. Our model predicts that CRH promotes bursting by decreasing the time constant of BK‐near and shifting the activation curve so that it becomes like BK‐far.

*In vivo*, global genetic deletion of BK channels results in mice with a blunted HPA axis response to acute stress, although the phenotype is probably manifest through changes at multiple levels of the HPA axis (Brunton *et al*. 2007). BK channels are expressed in several corticotroph models including murine corticotrophs where at least two splice variants, STREX and ZERO, are predominantly expressed (Shipston & Armstrong, 1996; Shipston *et al*. 1999; Brunton *et al*. 2007; Liang *et al*. 2011). CRH can inhibit total BK conductance in corticotrophs (Shipston *et al*. 1996; Tian *et al*. 2008) and in expression systems PKA‐dependent protein phosphorylation of either the STREX or ZERO variant results in channel inhibition or activation, respectively (Tian *et al*. 2001 *b*, 2004; Chen *et al*. 2005). More importantly, the properties of the STREX and ZERO variants correspond to the key features of BK‐near and BK‐far in the model, respectively, and a key effect of PKA phosphorylation of STREX is to convert it to a phenotype more closely corresponding to ZERO. Thus, a potential mechanism for CRH‐induced bursting may involve PKA‐dependent phosphorylation of STREX variant channels that may underlie the BK‐near conductance while also potentially enhancing BK‐far. Although PKC can also regulate BK channels, it can only regulate STREX variant channels under defined conditions, including channels that have already been phosphorylated by PKA (Zhou *et al*. 2012), suggesting that combined CRH/AVP may have an additional effect on BK‐near. Furthermore, as glucocorticoids have been shown to prevent PKA‐mediated regulation of BK channels (Shipston *et al*. 1996; Tian *et al*. 1998) it will be of interest to examine whether glucocorticoid feedback may control CRH‐induced bursting in native mouse corticotrophs. Clearly, alternative explanations for the molecular composition of these BK conductances are plausible and warrant future investigation.

Corticotroph cells treated with paxilline or isolated from BK^−/−^ mice showed no difference in spontaneous activity compared with controls, which suggests that BK channels are not responsible for regulating resting membrane potential or for the generation of spontaneous activity in corticotrophs. The model suggests that CRH/AVP promotes bursting primarily through BK channels and based on these observations one would predict that in the absence of BK channels, corticotroph excitability driven by CRH/AVP would result mostly in an increase in single‐spike frequency. Following CRH/AVP stimulation, both paxilline‐treated and BK^−/−^ cells showed an increase in event frequency but failed to significantly transition to bursting. In both cases bursting was not completely abolished, which suggests that although BK channels greatly facilitate bursting, they are not absolutely necessary. Indeed, this has been predicted in a previous modelling study of pituitary cells (Teka *et al*. 2011). Furthermore, bursting was more prevalent in BK^−/−^ cells compared to WT cells acutely exposed to the BK channel inhibitor paxilline, which could be the result of compensation through changes in expression of other ionic conductances. Previous studies have revealed that intermediate conductance Ca^2+^‐activated potassium (IK) channels may control bursting activity in female corticotroph cells (Liang *et al*. 2011), although the IK channel inhibitor TRAM‐34 has no effect in male corticotrophs. This suggests that male and female corticotrophs display sexually dimorphic mechanisms to control bursting.

In conclusion, we reveal that CRH and AVP regulate distinct patterns of electrical excitability in corticotrophs. Importantly, the CRH‐induced transition to bursting is dependent upon functional BK channels whereas AVP promotes an increase in spike frequency alone that is independent of BK channel function. The ability of these neuropeptides to engage distinct modes of electrical excitability is likely to have important functional consequences for the regulation of corticotrophs in response to different stressors.

## Additional information

### Competing interests

None of the authors have any competing interests to disclose.

### Author contributions

P.J.D., S.S., J.T., P.R., R.B. and M.J.S. designed the studies; P.J.D. performed electrophysiological recordings at the University of Edinburgh; S.S. and J.T. performed mathematical modelling at Florida State University; P.J.D., S.S., J.T., R.B. and M.J.S. analysed data; P.J.D., S.S., J.T., R.B. and M.J.S. wrote the paper. All authors have seen and approved the final version of the manuscript.

### Funding

P.J.D. was supported by an MRC PhD studentship in the College of Medicine and Veterinary Medicine, University of Edinburgh. Work was supported by grants to M.J.S. and P.R. from the Wellcome Trust (082407), to M.J.S. from MRC (J008893), and to R.B. and J.T. from the National Institutes of Health (DK43200).
